# Association of Zn and Cu Levels in Cord Blood and Maternal Milk with Pregnancy Outcomes among the Slovenian Population

**DOI:** 10.3390/nu14214667

**Published:** 2022-11-04

**Authors:** Joško Osredkar, Živa Miriam Geršak, Nataša Karas Kuželički, Janja Snoj Tratnik, Darja Mazej, Ingrid Falnoga, Milena Horvat, Ksenija Geršak

**Affiliations:** 1 Institute of Clinical Chemistry and Biochemistry, University Medical Centre Ljubljana, Njegoševa 4, 1000 Ljubljana, Slovenia; 2 Faculty of Pharmacy, Department of Clinical Biochemistry, University of Ljubljana, Aškerčeva 7, 1000 Ljubljana, Slovenia; 3 Faculty of Medicine, University of Ljubljana, Vrazov trg 2, 1000 Ljubljana, Slovenia; 4 Department of Environmental Sciences, Jožef Stefan Institute, Jamova cesta 39, 1000 Ljubljana, Slovenia; 5 Division of Gynaecology and Obstetrics, University Medical Centre Ljubljana, Slajmerjeva 3, 1000 Ljubljana, Slovenia

**Keywords:** zinc, copper, cord blood, maternal milk, gestational age, birth weight

## Abstract

Trace elements, including zinc (Zn) and copper (Cu), are known to play important roles in human health. The present study aimed to assess the levels of Zn and Cu in cord blood and maternal milk and to analyze their association with maternal and infant characteristics and pregnancy outcomes in a Slovenian study population of mothers and their neonates recruited within the PHIME prospective cohort study. The study included 324 mothers, but the data on Zn and Cu levels in both cord blood and maternal milk was available for 243 mothers. Questionnaires were used to assess the socio-demographic and health status of the mothers, their lifestyle habits (including detailed nutritional habits), and their residential and occupational histories. Inductively Coupled Plasma Mass Spectrometry (ICP-MS) was used to measure Zn and Cu levels in cord blood and maternal milk. Low Zn levels in cord blood were associated with lower gestational age and birth weight and were correlated with an increased probability of the birth of small for gestational age (SGA) infants. Maternal smoking influenced the Cu levels in both cord blood and maternal milk. Cord blood Cu levels were higher and Cu levels in maternal milk were lower in smoking compared to non-smoking mothers. Most importantly, a decreased Zn/Cu ratio in cord blood was associated with lower gestational age and lower birth weight. This indicates the overall positive effects of Zn and negative effects of Cu on pregnancy outcomes.

## 1. Introduction

Trace elements, including zinc (Zn) and copper (Cu), are known to play important roles in the maintenance of human health. Their role in several metabolic pathways, growth processes, cell development, and protein synthesis makes them crucial for human organ systems. Cu and Zn can take on different oxidation states, behaving as either electron acceptors or electron donors from their outer shell, providing them with the ability to act both as pro- and antioxidants, and why their concentration in the body is strictly regulated [1,2,3]. Furthermore, both a deficiency in and an excess of Cu and Zn have been shown to cause oxidative stress. The relationship between the concentration of these trace elements in the body and the level of oxidative stress is defined as U-shaped [4,5,6,7].

Zn is the second most abundant trace element in the human body and plays an important role in the metabolism of proteins, carbohydrates, and lipids. Additionally, it is essential for cellular differentiation and replication [8,9], and is required for the catalytic activity of more than 200 enzymes; in fact, it is the only metal that appears in all enzyme classes [10]. Cu is the third-most abundant trace element and a crucial component of several essential enzymes named cuproenzymes. These enzymes catalyse important physiological reactions related to oxidative phosphorylation, the deactivation of free radicals, iron metabolism, catecholamine synthesis, and more [11]. In short, Zn and Cu, both essential micronutrients, are required for a wide variety of enzymatic and other processes and have significant prominence, particularly during embryonic and foetal development. 

A sufficient maternal zinc concentration is essential for embryogenesis, since the most important effect of zinc is to regulate the structure and function of chromatin and thus gene expression [12]. Consequently, insufficient zinc levels during embryogenesis can affect the final phenotype of all organs. Fetal death, growth retardation, and various malformations including neural tube defects are present at an increased rate when zinc deficiency is involved [13,14,15,16]. Zinc concentrations increase with the duration of pregnancy, most markedly after 24 weeks of gestation [17]. The mechanism of zinc transfer across the placenta to the fetus is mainly by endocytosis, which is then stored in the fetal liver [18,19]. Zinc deficiency in hospitalized preterm neonates has been well documented [20]. It may be linked to the confluence of several risk factors, i.e., low body stores due to the shorter time for placental transfer of zinc, likely reduced uptake, and increased endogenous losses. We have a large body of evidence that a large percentage of preterm neonates have mild to moderate subclinical zinc deficiency, and the consequences of zinc deficiency in preterm infants vary widely and in many cases can be severe [21]. The consequences of zinc deficiency in pregnancy are primarily impaired immunity, followed by prolonged labor, preterm or late labor, intrauterine growth retardation, low birth weight, and pregnancy-induced hypertension [22,23].

The concentrations of nutrients in the human placenta and milk are of great interest. The placenta provides an essential source of nutrients of for fetal development, and maternal milk is the first and the only source of food for infants in the neonatal period, wherefore it should meet all the neonate’s metabolic demands during its first three months of life [24,25]. 

Micronutrients Zn and Cu have to be transported from the maternal circulatory system and, before reaching the child, have to cross two barriers: the placenta, acting as a membrane between maternal and fetal blood, and the second barrier, which is formed by the mammary gland and is between maternal blood and the mother’s milk [24]. Several studies have reported that Cu levels in maternal serum are much higher than Cu levels in fetal serum, indicating Cu mobilization in the mother during pregnancy [26,27]. The great majority of serum Cu is transported bound to ceruloplasmin, however, the low concentration of ceruloplasmin in the serum of neonates indicates that ceruloplasmin cannot cross the human placenta, pointing to the fact that the placenta has a blocking effect on the transfer of Cu from the mother to the fetus. Placental Cu uptake is performed through a high-affinity carrier (Ctr1) expressed early in the pregnancy [28], which is responsible for the transport of 80% of Cu and other metals into cells [29,30]. Once taken up by the placenta, it is bound to one of a series of chaperone proteins and carried to the ATPase, where it is pumped across the cell membrane [31]. Antioxidant protein 1 (ATOX1) transports Cu ions to the P-type Cu transport ATPase ATP7A and the β-type Cu transport ATPase (ATP7B), which regulate the concentration of Cu ions in the cell and mediate the incorporation of Cu cations into enzymatic proteins [32]. Cu uptake by the mammary gland is the same as in the placenta; mediated by the Ctr1 transporter. Petris et al. showed that Ctr1 is vesicular; it is endocytosed and proteolytically degraded in response to increased Cu levels, thereby providing a means of regulating cellular Cu uptake [33]. Cu concentration in maternal milk is actually a fraction of that in maternal serum [34].

In contrast to Cu, the Zn level in cord blood serum is consistently higher than the corresponding levels in maternal blood [35]. Furthermore, lactic Zn concentration is significantly higher than in maternal serum, at least for the first several months of lactation, and, besides that, the transfer into milk is almost twice as high as the Zn transfer across the placenta during late pregnancy [34,36]. In vitro studies of human placenta and animal models showed that placental Zn uptake and release involve an active process [37,38]. Zn carriers are metalloproteins specialized in the selective capture and transport of Zn ions across the membrane barrier. In this two-step molecular process, the chemical properties of Zn itself determine the composition and geometry of the metal binding sites and, in their immediate surroundings, allow for the selectivity of Zn binding [39]. In the human placenta, ZnT1-8 and ZIP1 were detected in BeWo cells [40], and ZnT1 and ZnT5 were detected in villous syncytiotrophoblast cells [41] as the major factors involved in Zn homeostasis regulation. Although Zn transporter expression has been measured in the human placenta, the regulatory role of this transporter in the transfer from the mother to the fetus is unknown [42]. Zinc uptake and transfer into milk is facilitated by the α-2-macroglobulin receptor in the mammary gland’s epithelial cells. A role in regulating milk Zn concentration throughout lactation is also played by Zn importers, such as ZIP3, and Zn exporters, such as ZnT-1, ZnT2, and ZnT4, which are localized in the alveolar lumen and other cells [34,37,43]. 

Insufficient levels of Cu and Zn may promote oxidative stress by impairing antioxidant mechanisms, while an imbalance of these two trace elements may be closely associated with complications during pregnancy, including preterm delivery, intrauterine growth restriction, congenital malformations, premature rupture of membranes, and other adverse pregnancy outcomes [14,44,45,46]. Their deficiency may occur during pregnancy due to low maternal dietary intake, disease-induced or drug-induced changes in maternal and embryonic Zn/Cu metabolism, or due to some other mechanisms [14,47]. The possible consequences of excessive or deficient Cu and Zn concentrations during pregnancy are shown in Table 1.

However, excess metals in milk can cause changes in the structure and function of the immune system by disrupting homeostasis. These inorganic elements can stimulate or suppress immunomodulatory components, thus indirectly affecting various bodily organs and the nervous, reproductive, respiratory, and endocrine systems. This in turn can cause various health problems in children. Allergies have been described, and endocrine disruption and even neurodevelopmental delays and disorders can occur [48].

The Cu/Zn ratio provides a better insight into the situation than measuring Zn and Cu alone. It is the ratio that indicates a reduced ability to maintain or re-establish homeostasis after a destabilizing event [49]. Scientific literature shows that the ratio is mainly related to inflammatory mediators, while there is no association with dietary factors [50]. High Cu/Zn ratios are found in chronic inflammatory diseases [51] and increased oxidative stress coexisting in patients with chronic diseases [52].

The present study aimed to assess the levels of Zn and Cu in cord blood and maternal milk, and to analyze their association with maternal and infant characteristics and pregnancy outcomes in a Slovenian study population of mothers and their neonates. It is a continuation of the work under the overall objectives of the PHIME, focusing on Cu and Zn as two of the most important micronutrients in early life and on their role in children’s normal development. 

## 2. Materials and Methods

### 2.1. Birth Cohort

The birth cohort was established to study long-term mixed element exposure in prenatal life and its association with health outcomes, primarily the neuropsychological development of children. Studies focusing on prenatal mercury exposure have been published before by Miklavčič et al. (2011, 2013) [53,54], Snoj Tratnik et al. (2017) [55], Barbone et al. (2019) [56], and Trdin et al. (2020) [57].

### 2.2. Study Design

This prospective cohort study was set within a 5-year integrated project on the public health impact of long-term, low-level, mixed element exposure in susceptible population strata (PHIME) as part of the Mediterranean cohort study, involving study populations from Slovenia, Croatia, Italy, and Greece. The present study includes the recruitment area of the city of Ljubljana, Slovenia, and its surroundings (up to 50 km). The study design, including the setting, recruitment, criteria for exclusion, questionnaires, biological sampling, and outcome assessment are described in detail by Valent et al. (2013) [58]. In short, women eligible for recruitment were approached for consent during their maternity ward stay for delivery. Recruitment took place at the Maternity Hospital of the University Medical Centre of Ljubljana. The National Ethics Committee of Slovenia approved the study (No. of accordance 98/05/06, date of issue 16 May 2006). 

### 2.3. Questionnaire

The study utilized two questionnaires: one short (35 general questions) and one long (82 questions).

Pregnant women (between their 27th and 32nd weeks of pregnancy) who agreed to participate in the study were given a short questionnaire by their gynecologists. The long, detailed questionnaire was completed one month after delivery in order to obtain information on maternal demographics and health status, lifestyle (including detailed nutritional) habits, and residential and occupational history. The long questionnaire is included in the Supplements of this article (Questionnaire S1).

### 2.4. Definition of Size for Gestational Age, Gestational Age

In this study, small for gestational ages (SGA) births were defined as live-born infants that were <10th percentile of birth weight (at 40 weeks <3020 g for boys and <2889 g for girls), appropriate for gestational age (AGA) births were defined as 10–90th percentile of birth weight, and large for gestational age (LGA) births were defined as >90th percentile of birth weight (at 40 weeks >4121 g for boys and >3921 g for girls) according to nomograms based on sex and gestational age from the total Slovene cohort of singleton infants (N = 223,949) [59]. 

The study group was also subdivided according to gestational age into pre-term (<37 weeks), term (37–42 weeks), and post-term (>42 weeks) groups.

### 2.5. Collection of Cord Blood and Maternal Milk Samples

We employed boxes with collection tubes that were additionally labelled with a code number, date, and sample type. The sampling of mixed umbilical cord blood was done immediately after delivery in an obstetrical room. Whole blood was collected in 7-mL tubes (NaH) for elemental determination. Aliquoting and distribution were performed in the hospital laboratory; subsequently, the aliquots were frozen (below −24 °C) and then transported to the Jožef Stefan Institute’s (JSI) laboratory for elemental determination. Breast milk was collected 6–8 weeks after delivery by the mother and transferred to the JSI laboratory, where it was stored at −20 °C for subsequent analysis of element content.

Whole cord blood concentrations of Cu were used as the study lacks determination in serum or plasma, even though their levels are usually used as an indicator of an essential element’s status, presenting an exchangeable, biologically relevant fraction, while blood cells act as a storage compartment. However, in the matter of Cu, both compartments–plasma with ceruloplasmin bound Cu and red blood cells with Cu mostly bound to SOD1–present similar concentrations of this element [60]. Whereas for Zn, there is essentially no single biochemical criterion that can reliably reflect Zn body stores [61], since Zn is a component of many proteins and nucleic acids, and cellular functions [60,62]. Zn levels in serum/plasma are still widely used to assess Zn status, but combining these with other measures (e.g., urine Zn levels, erythrocyte metallothionenin levels, symptoms) is recommended for a more accurate determination of the status [61]. Considering the above-listed knowledge, we hypothesized that inter-individual changes in Cu and Zn status could also be reflected in whole blood levels, as there already are some reports available observing differences in whole blood levels between autistic children and controls [63].

### 2.6. Measurement of Zn and Cu in Cord Blood 

An aliquot of 0.3 mL of blood sample was diluted ten times with an alkaline (Merck, suprapur) solution containing Triton X-100 (Sigma Aldrich, MA, USA, sigma ultra) and ethylenediaminetetraacetic acid disodium salt dehydrate (EDTA, Fisher Scientific, MA, USA, analytical reagent grade) [64]. An aliquot of an internal standard solution containing Ga, Gd, Y, and Sc was added. For calibration, the standard addition procedure was performed. Measurements of prepared solutions were made by an Inductively Coupled Plasma Mass Spectrometer (ICP-MS, 7500ce, Agilent, Tokyo, Japan) equipped with an Octapole Reaction System (ORS) and an ASX-510 Autosampler (Cetac). Instrumental conditions were as follows: Babington nebulizer, Scott-type spray chamber, spray chamber temperature at 5 °C, plasma gas flow rate 15 L/min, carrier gas flow rate 0.8 L/min, make-up gas flow rate 0.1 L/min, sample solution uptake flow rate 1 mL/min, RF power 1500 W, reaction cell gas helium 4 mL/min in ORS to avoid interference, and isotopes monitored 55Mn, 63Cu, 66Zn, 75As, 78Se, 111Cd, 114Cd, 206–208Pb. The instrument was recalibrated daily. To ensure quality, blank samples, control samples, and reference materials were run together with the samples on a daily basis. The analytical precision was 5%. The limits of detection (LOD), calculated as three times the standard deviations of the blank sample, were 7 µg/L for Cu and 30 µg/L for Zn. A reference material Seronorm^®^ (Trace Elements in Whole Blood L-1, SERO AS, Billingstad, Norvay) was used to check the accuracy of the results [65].

### 2.7. Measurement of Zn and Cu in Maternal Milk

Milk samples were prepared for analysis using microwave digestion. These procedures are described in detail in Potočnik et al., 2016 [66]. In brief, about 1 mL of milk sample (0.15 g of lyophilized sample) was poured into quartz tubes. Then, 1 mL of 65% HNO3, (Merck, Darmstadt, Germany, suprapur) and 1 mL 30% H2O2, (Merck, Germany, suprapur) were added, and the samples were subjected to closed vessel microwave digestion (Microwave system ETHOS 1, MILESTONE Srl, BG, Italy) at a max. power of 1500 W. The procedure was a temperature ramp to 130 °C in 10 min., ramp to 200 °C in 10 min., hold for 20 min., and cool for 20 min. Then, the samples were equilibrated to room temperature. The solution was quantitatively transferred to a polyethylene graduated tube and filled to 10 mL with Mili-Q water. External calibration was made using ICP Multi Element Standard solution XVI CertiPUR (Merck, Germany).

To ensure quality, blank samples, control samples, and reference materials were run together with the samples daily. Analytical precision was 5% for both elements. LODs, calculated as described above were 6 µg/L for Cu and 35 µg/L for Zn milk sample, respectively. The accuracy of the results was verified by analyzing the certified reference and control materials Whole milk powder NIST 8435, Non-fat Milk Powder NIST 1549 (both National Institute of Standard and Technology, NIST, Gaithersburg, MD, USA) and Skim Milk Powder BCR 150 (EC-JRC-IRMM, Geel, Belgium), and Whole milk powder NIST 1549a (NIST, MD, USA) and FAPAS 7190/7172 (Proficiency testing from Fera Science Ltd., York, UK). As there are no suitable reference materials, the results of the laboratory control milk sample were compared by an independent method, namely radiochemical neutron activation analysis. The quality control procedures for milk samples are described in detail in Potočnik et al., 2016 [66].

### 2.8. Statistical Analysis

The normality of the data distribution was evaluated by the Kolmogorov–Smirnov test. Measurements with Gaussian distribution were analyzed using one-way ANOVA (to compare 3 or more unmatched groups) and Pearson correlation (to quantify the association between two Gaussian variables). Measurements with non-Gaussian distribution were analyzed using the Kruskal–Wallis test (to compare 3 or more unmatched groups) and Spearman correlation (to quantify the association between two non-Gaussian variables). To compare binomial data, Fisher’s exact test was used. A level of confidence of α = 0.05 was considered statistically significant. The data were adjusted to the confounding variables using multiple regression for continuous outcome variables and multinomial logistic regression for categorical outcome variables. Collinearity diagnostics were conducted to avoid multiple collinearity by evaluating correlation coefficients in the covariance matrix and variance inflation factor (VIF) values, where correlation coefficients less than 0.8 and VIF values around 1 were considered appropriate.

## 3. Results

### 3.1. Normality of Data Distribution

Most of the non-categorical data were non-normally distributed, except for infant birthweight, pregnancy BMI increase, and Cu and Zn breastmilk levels, which showed a normal distribution.

### 3.2. Association of Maternal Characteristics with Cu and Zn Levels in Cord Blood and Maternal Milk

The study included 324 mothers, but data on Zn and Cu levels in both cord blood and maternal milk was only available for 243 mothers. The mean maternal age at delivery, pre-pregnancy BMI, and pregnancy BMI increase were 30.1 ± 4.0 years, 23.6 ± 4.2 kg/m^2^, and 5.13 ± 1.80 kg/m^2^, respectively. The majority (59.9%) of mothers had a university degree, were employed (88.3%), and were married or in a committed relationship (97.6%). Maternal demographics are described in detail in Table 2.

The mean maternal milk concentrations of Cu and Zn were 556 ± 142 µg/L and 3195 ± 1428 µg/L and median concentrations of Cu and Zn in whole cord blood were 557 (min: 313, max: 2623) µg/L and 1780 (min: 745, max: 10,964) µg/L, respectively.

Simple (bivariate) statistical analysis showed no significant relationship between maternal age at delivery, socio-economic status, marital status, and maternal pre-pregnancy BMI and Zn and Cu levels in maternal milk or cord blood. On the other hand, a greater maternal BMI increase during pregnancy was associated with lower levels of Cu in cord blood (Spearman’s rho = −0.132, *p* = 0.017), and maternal education with Cu and Zn levels in maternal milk.

We also compared Cu and Zn levels in cord blood and maternal milk with respective reference intervals [67,68] across maternal characteristics. For Cu and Zn in cord blood gestational age-specific reference intervals are presented in Table 3. References in intervals in maternal milk were 117–645 µg/L for Cu and 1455–8355 µg/L for Zn. 

**Figure 1:**
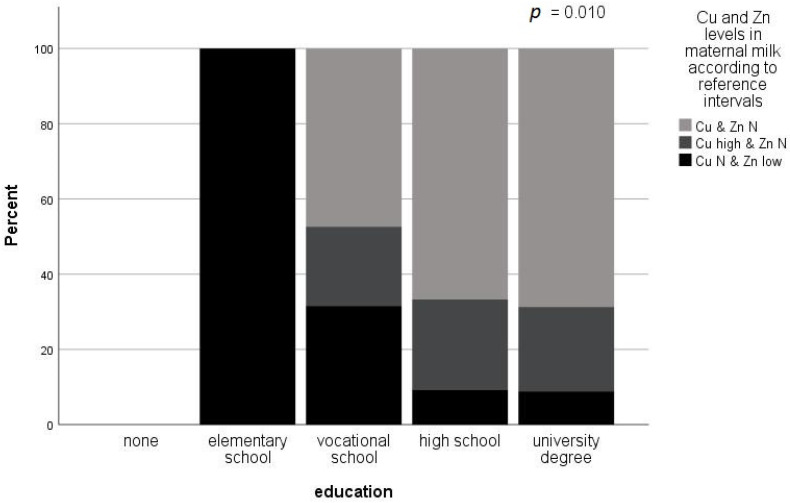
Influence of maternal education on Cu and Zn levels in maternal milk according to reference intervals (Cu: 117–645 µg/L, Zn: 1455–8355 µg/L) (Fisher’s exact test). Maternal education was associated with Zn and Cu levels in breast milk. A higher level of education was associated with normal (within reference intervals) levels of Zn and Cu in maternal milk, while a lower level of education was associated with low Zn levels in maternal milk. High Cu levels in maternal milk were not associated with maternal education.

A lower level of education was associated with low Zn levels, while a higher level of education was associated with normal levels of Zn and Cu in maternal milk (Fisher’s exact *p* = 0.010). The results are presented in Figure 1.

**Figure 2:**
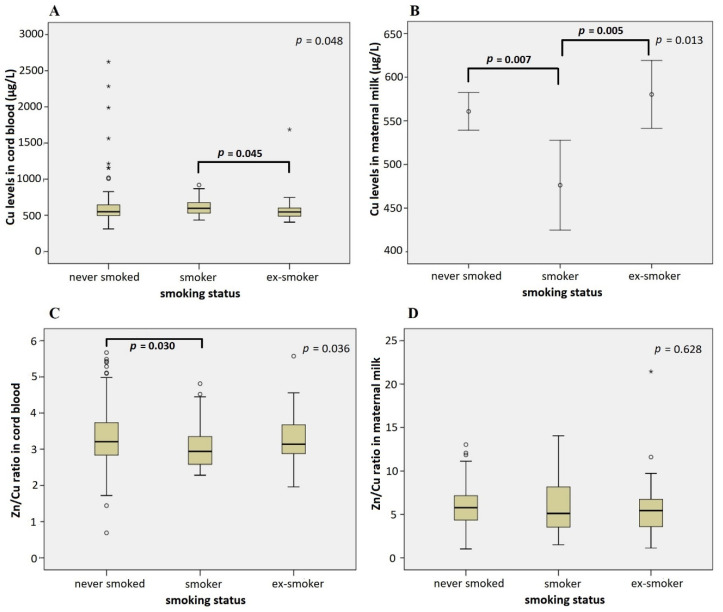
Influence of the maternal smoking status on Cu levels in cord blood (**A**), Cu levels in maternal milk (**B**), the ratio of Zn/Cu levels in cord blood (**C**), and the ratio of Zn/Cu levels in maternal milk (**D**) (Kruskal–Wallis test (**A**,**C**,**D**) and One-way ANOVA (**B**)). Significant differences between subgroups are denoted using *p* values of post hoc analysis. Circles denote outliers and asterisks denote extreme outliers.

Furthermore, we found an association between both micronutrients in both studied compartments and maternal smoking status. Children of smoking mothers had significantly higher Cu cord blood levels (612 ± 15.6 µg/L) than children of ex-smokers (571 ± 22.5 µg/L) and mothers that never smoked (606 ± 16.6 µg/L) (*p* = 0.048). The association of maternal smoking with Cu levels in maternal milk was just the reverse; statistically significant lower Cu levels in maternal milk were identified in smoking mothers (476 ± 24.8 µg/L) compared to ex-smokers (580 ± 19.3 µg/L) and mothers that never smoked (561 ± 10.9 µg/L) (*p* = 0.013) (Figure 2). Post hoc comparison revealed no differences in Cu cord blood and maternal milk levels between the infants of ex-smokers and mothers that never smoked.

In addition to Cu levels in both studied compartments, smoking status was also associated with the Zn/Cu ratio in cord blood (Figure 2). Smoking mothers had a lower Zn/Cu ratio in cord blood than ex-smokers and mothers that never smoked (*p* = 0.036).

### 3.3. Association of Infant Characteristics and Pregnancy Outcomes with Cu and Zn Levels in Cord Blood and Maternal Milk 

The study involved 159 male and 165 female infants, who were mostly born by spontaneous (46.3%) or induced (46.3%) vaginal delivery. The mean gestation age, birth weight, and birth length of the infants were 39.52 ± 1.66 weeks, 3439.10 ± 486.47 g, and 51.75 ± 2.30 cm, respectively (Table 4).

A simple statistical analysis showed no significant relationship between sex of the infant, type of delivery, and micronutrient contents in maternal milk or cord blood. On the other hand, there were statistically significant differences in Cu and Zn contents in maternal milk and cord blood according to gestational age, birth weight, and birth height. 

**Figure 3:**
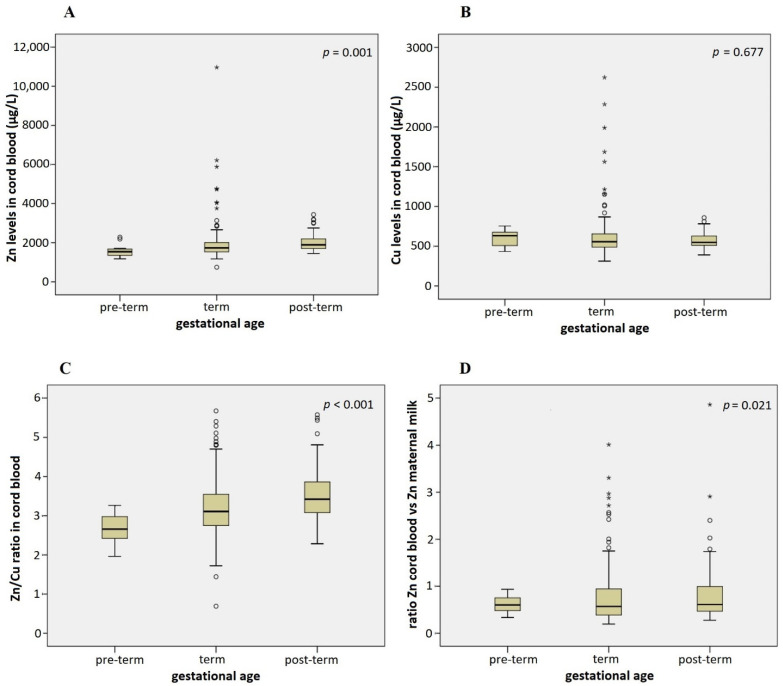
Association of infant gestational age with Zn levels in cord blood (**A**), Cu levels in cord blood (**B**), Zn/Cu ratio in cord blood (**C**), and the ratio between Zn levels in cord blood and maternal milk (**D**) (Kruskal–Wallis test). Adjusted for maternal age, sex of the infant, smoking status, BMI increase in pregnancy, and marital status in multiple regression models: *p* = 0.014 (**A**), *p* = 0.656 (**B**), *p* < 0.001 (**C**), *p* = 0.244 (**D**). Circles denote outliers and asterisks denote extreme outliers.

Gestational age was in positive correlation with Zn levels in cord blood; infants reaching the higher gestational age have higher Zn levels in cord blood compared to younger infants (Spearman’s rho = 0.333, *p* < 0.001). This association remained significant after adjusting for maternal age, sex of the infant, smoking status, BMI increase in pregnancy, and marital status (multiple regression *p* = 0.014). Pre-term infants, born before 37 completed weeks of gestation, had the lowest levels of Zn in cord blood (1592 ± 107 µg/L), and post-term infants, born after 42 weeks, had the highest Zn levels (2003 ± 40.3 µg/L) (Figure 3). Children born within term (37–42 weeks) had intermediate levels of Zn in cord blood (1910 ± 62.0 µg/L), but post hoc comparison showed no statistical difference in Zn cord blood levels between pre-term and term infants, indicating that post-term birth is associated with increased Zn levels in cord blood (Figure 3A). 

Pre-term infants had a lower Zn/Cu ratio in cord blood than term and post-term infants (2.7 vs. 3.2 and 3.5, *p* < 0.001) (Figure 3C), and post-term infant/mother pairs had a higher ratio between Zn levels in cord blood and maternal milk compared to term and pre-term infant/mother pairs (0.8 vs. 0.6 and 0.5) (Figure 3D). The first association remained significant in the multiple regression model (*p* < 0.001), while the second one disappeared after the adjustment (*p* = 0.244). No statistically significant differences were noted for Cu levels in cord blood according to the gestational age.

**Figure 4:**
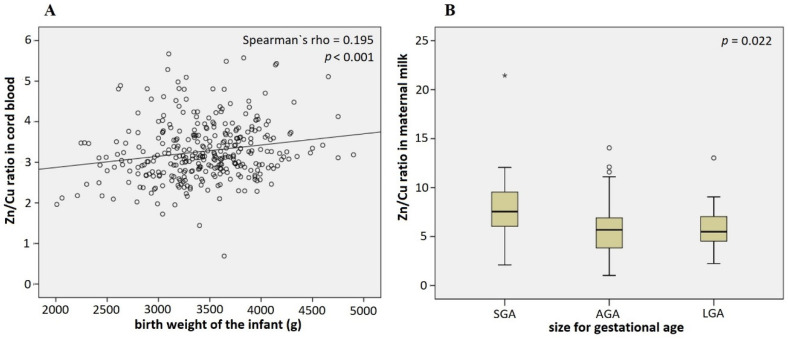
Association of the infant birth weight with Zn/Cu ratio in cord blood (**A**) and in maternal milk (**B**) (Spearman’s rho (**A**) and Kruskal–Wallis (**B**) tests). Adjusted for maternal age, sex of the infant, smoking status, BMI increase in pregnancy, and marital status in the multiple regression model: *p* = 0.001 (**A**) and the multinominal regression model: AGA vs. SGA *p* = 0.002, AGA vs. LGA *p* = 0.468 (**B**). Circles denote outliers and asterisks denote extreme outliers.

In addition to gestational age, infant birth weight was also found to be positively correlated to Zn levels in cord blood (Spearman’s rho = 0.112, *p* = 0.043, adjusted in the multiple regression model *p* = 0.072) and the Zn/Cu ratio in cord blood (Spearman’s rho = 0.195, *p* < 0.001) (Figure 4A). Birth weight was associated with Zn/Cu ratio in cord blood even after the adjustment for confounding variables in the multiple regression model (*p* = 0.001). 

To avoid the confounding effect of correlation between gestational age and birth weight on the results, we also analyzed the data considering size for gestational age. Size for gestational age is a measure of fetal growth, considering epidemiological data of gestational age-appropriate infant birth weight; infants are categorized into three groups accordingly: (1) small for gestational age (SGA), (2) large for gestational age (LGA), and (3) appropriate for gestational age (AGA). In this study, we demonstrated that mothers of SGA infants had higher Zn/Cu ratios (7.5) in maternal milk than mothers of AGA (5.7) and LGA (5.5) infants (*p* = 0.022) (Figure 4B). This was confirmed after adjusting for maternal age, sex of the infant, smoking status, BMI increase in pregnancy, and marital status in the multi-nominal logistic regression model (AGA vs. SGA *p* = 0.002, AGA vs. LGA *p* = 0.468). No such association was present for Zn/Cu ratio in cord blood (*p* = 0.145). 

A low but statistically significant positive correlation was detected between Zn levels in cord blood and infant birth length (Spearman’s rho = 0.143, *p* = 0.010). Furthermore, a statistically significant positive correlation was also detected between Zn/Cu ratios in cord blood and birth length of the infant; larger infants had higher Zn/Cu cord blood ratios than smaller infants (Spearman’s rho = 0.188, *p* = 0.001).

## 4. Discussion

The present study aimed to assess the levels of Zn and Cu in maternal milk and cord blood and to analyze their association with maternal and infant characteristics in the Slovenian population. 

Breast milk has three different and distinct stages; colostrum is the first form of milk secreted in the 2–3 days after delivery, transitional milk occurs from day 7 to 14, and mature milk after 2 weeks post-delivery, and the content of trace elements varies in each stage [69]. The mean maternal milk Cu and Zn levels in Slovenian mothers were 556.14 ± 142.26 µg/L and 3195.14 ± 1427.50 µg/L, respectively. 

Copper values found in maternal milk samples in our study were somewhat higher than the levels reported by Ellingsen et al. [60] in mature milk, 1–6 months after delivery (mean 290 µg/L, range 90–600 µg/L). The levels were also higher than those reported previously for the mature milk of 7 Slovenian mothers (mean 247 µg/L, range 20–470 µg/L) [70]. Accordingly, there were only 3 mothers with a Cu level below the lower reference value of 200 µg/L, while most of the mothers’ (n = 218; 87%) milk levels were above the upper reference value of 400 µg/L set by Iyengar [71]. Similar results were observed for Zn milk levels, with 12 mothers below the lower reference level (1000 µg/L) and 192 (76%) above the upper reference level (2000 µg/L) [71], which means that excessive levels of both elements were more frequent than insufficient levels. In comparison to the levels determined previously in the mature milk of Slovene mothers (mean 1400 µg/L, range 310–3040 µg/L, n = 7) [70], the levels in the present study were considerably higher. According to the national reference values set for the mature milk of a representative population of primiparous lactating women sampled between 2008 and 2014 across Slovenia, the majority of mothers in the present study had their Cu and Zn levels within the established intervals; however, 36% of mothers had Cu levels above the upper reference level of 600 µg/L [72], while 3 were below. For Zn, there were 2 women below the lower reference value of 600 µg/L and 20 above the upper reference value of 5000 µg/L [72]. 

The mean Cu and Zn levels in cord blood obtained in our study were 600 ± 220 µg/L (range 388–1388 µg/L) and 1928 ± 775 µg/L (plasma Zn: 936 ± 155 µg/L, range 595–1432), respectively. A recently published study including 53 Spanish mother-infant pairs reported similar levels for Cu (median 623 µg/L, range 386–813 µg/L) and Zn (median 2311 µg/L, range 1489–3049 µg/L) in whole cord blood [73]. Normally, essential element concentrations are reported in serum, wherefore only a few comparisons were possible for our study population. However, our study determined plasma concentrations of Zn alongside cord blood levels, which was 936 ± 155 µg/L (range 595–1432 µg/L) and was in agreement with the levels previously observed in Slovene neonates born in 1996–97 in Maribor (930 ± 1220 µg/L, range 610–1680 µg/L) [18], while slightly higher than in Indian neonates’ serum (887 ± 98 µg/L, n = 80) [40], and somewhat lower than in neonates from Jordan (1140 ± 230 µg/L, n = 92) [74].

The above-listed comparisons should be interpreted with caution, as the levels in milk have not been normalized per water content and are expressed per volume of milk, and the levels in cord blood are reported for whole blood instead of serum or plasma. Moreover, mixed cord blood was sampled containing both venous blood, which is enriched with all necessary nutrients from the maternal blood and arterial blood from two arteries that have passed the baby and circulate back to the placenta to take up nutrients again [5]. However, even with these two limitations, our results indicate that maternal milk levels are excessive, rather than deficient, in both elements. The present study observed no significant relationship between most maternal characteristics and Zn and Cu levels in maternal milk or cord blood. However, greater maternal BMI increase during pregnancy was associated with lower levels of Cu in cord blood. The same correlation between serum Cu levels and BMI was observed in a study on male patients with androgenic alopecia [75]. A few studies have found a positive association between Cu and serum lipids [76,77], while others have found inverse relationships [78]. However, we must take into account that the average pregnancy increased weight gain (12.5 kg) [79] is due to the products of conception (fetus, placenta, and amniotic fluid, approximately 5.0 kg) and changes in the mother (enlarged uterus and breast tissue approx. 1.4 kg, and extracellular extravascular and intravascular fluid approx. 3000 mL) and not necessarily due to the accumulation of fat (approximately 3.5 kg), therefore, it is not possible to undoubtedly connect greater BMI with obesity, leptin, or other mechanisms [80].

In addition, maternal education was identified as the characteristic that was associated with Cu and Zn levels in maternal milk, where a lower level of education was associated with low Zn levels, while a higher level of education was associated with normal levels of Zn and Cu in milk. Diet is the main factor that determines Zn status and in many countries, such as Australia or the United States, Zn supplementation is recommended for pregnant women [81,82]. It is widely acknowledged that many pregnant women do not meet this recommendation, which might be at least partially attributed to lower maternal education levels. 

According to this study, maternal smoking status was, among maternal characteristics, the major determinant of Cu status in cord blood and maternal milk (Figure 2). Cigarette smoking was found to contribute to an increase in the plasma concentrations of cadmium, lead, and copper, while it had no influence on plasma Zn concentration and is recognized as a significant source of oxidative stress [83,84]. Although the placenta is the essential interface between maternal and fetal circulation that normally maintains the proper balance of the fetus’s metabolic needs, it can be irreversibly damaged by oxidative stress and consequently stop functioning properly. The presence of a disrupted placenta barrier due to oxidative stress may contribute to excessive Cu transport from maternal blood to fetal circulation, resulting in the increased Cu cord blood levels, which was observed in this as well as in other studies [85,86]. According to the present study, smoking mothers also had a lower Zn/Cu ratio in cord blood compared to ex-smokers and mothers that never smoked. Dore [86] showed that altered Cu metabolism due to smoking did not affect Cu levels in colostrum, while Leontiniids al. [68] and the present study showed that smoking mothers had lower Cu levels in colostrum/mature milk compared to ex-smokers and mothers that never smoked. Interestingly, both high Cu cord blood levels and maternal smoking were associated with decreased birth weight [85]. What is more, in addition to increased levels of Cu in cord blood of smoking mothers, we also observed a higher incidence of SGA infants in smoker and ex-smoker mothers compared to those mothers who never smoked (Fisher exact test, *p* = 0.026).

A few more statistically significant differences in the Cu and Zn content of maternal milk and cord blood were found according to infant characteristics. A statistically significant positive correlation was found between Zn levels in cord blood and gestational age. This correlation was in agreement with previous studies [87,88,89] and can be explained by the fact that the majority of fetal Zn accumulation occurs during the last trimester of gestation [78]. King suggested that absorption of Zn increases by 30% in late pregnancy [90]. There was no correlation between gestational age and Cu concentration in cord blood in the present study and by others [91]. However, the Zn/Cu ratio in cord blood correlated positively with gestational age in the present study.

Preterm infants commonly have a low birth weight and are smaller, so it is not surprising that this study observed birthweight as marginally positively correlated to Zn levels in cord blood. Other studies showed similar results; mothers of low-birth-weight neonates had significantly lower serum Zn levels than mothers who gave birth to normal birth weight neonates; there was a positive correlation between maternal Zn level and birth weight [46,92]. What is more, in the present study the statistically significant positive correlation was demonstrated between birthweight and Zn/Cu ratio in cord blood, which confirms the negative effect of high Cu levels on fetal growth observed by others [85].

To avoid the confounding effect of correlation between gestational age and birth weight on the results, we also analyzed the data taking into account size for gestational age. According to this, we could not confirm a positive association between Zn levels or Zn/Cu ratio in cord blood and birth weight, however, we demonstrated that mothers of SGA infants had a higher Zn/Cu ratio in maternal milk than mothers of AGA and LGA infants, which we are the first to report, to the best of our knowledge.

This is an important finding; particularly as preterm and SGA infants have higher essential micronutrient requirements due to their rapid postnatal growth and development and the limited capacity for storage of these elements [93]. Therefore, these infants are at an increased risk of developing nutritional deficiencies. Upstanding et al. reported significantly lower Zn levels in milk from mothers of pre-term versus full-term babies [94], however, our study showed the opposite, indicating that higher micronutrient requirements of infants born with low birth weight for gestational age might be compensated through higher Zn levels in maternal milk in comparison to infants who were born with normal or higher weight according to gestational age.

To date, benefits of zinc nutriture during pregnancy have not been demonstrated and as zinc deficiency is likely a reflection of poor diet, strategies to improve overall maternal nutrition are likely to yield more tangible health benefits than the use of zinc supplements alone [95].

The results of the present study allow us to reach the following conclusions. Low Zn levels in cord blood were associated with lower gestational age and birth weight and were correlated with an increased probability of the birth of small for gestational age (SGA) infants. Maternal smoking influenced the Cu levels in both cord blood and maternal milk. Cord blood Cu levels were higher and Cu levels in maternal milk were lower in smoking compared to non-smoking mothers. Most importantly, decreased Zn/Cu ratio in cord blood was associated with lower gestational age and lower birth weight. Cu/Zn in whole blood reference range is 0.12–0.17, but the range for our samples was 0.19–0.43. This indicates an overall positive impact of Zn and negative impact of Cu on pregnancy outcomes. 

This is in line with Zn’s known antioxidant properties and Cu’s prooxidant properties. Cu exerts its prooxidative influence mostly through the Haber–Weiss reaction [96], which generates hydroxyl radicals, the most powerful reactive oxygen species (ROS) arising in biological systems. Zn, on the other hand, functions as an antioxidant mostly through the competitive antagonism of iron and Cu prooxidative action, and through the activation of antioxidant proteins, molecules, and enzymes, such as glutathione, catalase, superoxide dismutase, metallothionein, and others [97]. Thus, a decreased Zn/Cu ratio could be associated with increased levels of ROS, which negatively affects pregnancy outcomes [98].

Our cohort sample represents a population from the central part of Slovenia (Ljubljana and its surroundings) living in a relatively uniform environment, and the sample size was sufficiently large to enable comparison of Cu and Zn levels between different groups of the study population. The already mentioned limitations of the study include non-normalized milk levels of Cu and Zn; and lack of measurements of both Cu and Zn in serum or plasma of the venous cord blood, which would enable more accurate determination of status, particularly concerning ratio.

Zinc concentrations in human milk vary among geographical regions but are not associated with maternal blood concentrations. Copper is highly bioavailable, and its concentration in human milk is also not associated with maternal blood concentrations. Neonates have large liver stores, which protect them against deficiency in early life.

In conclusion, this study provides valuable information on Cu and Zn cord blood and maternal milk levels in neonates and mothers in the Slovenian population, as well as insights into the influence of adequate Zn and Cu concentrations on positive pregnancy outcomes.

## Figures and Tables

**Table 1:** Effects of Cu and Zn on pregnant women [14,44,45,46].

Intake/Concentration	Cu	Zn
**Excess**	Preterm birth Low birth weight Gestational diabetes	Neuronal defects Teratogenic Lethal
**Deficiency**	Underdevelopment of nervous system Intrauterine growth restriction Spontaneous delivery Spontaneous abortion	Preterm birth Pregnancy induce hypertension Low birth weight Preeclampsia Placental insufficiency
**Normal range in whole blood**	508–1307 µg/L (8.0–20.6 µmol/L)	4290–7600 µg/L (66–116 µmol/L)

**Table 2:** General characteristics of mothers (n = 324).

Variable	Mean ± SD or Frequency (%)
**Age at delivery (years)**	30.05 ± 4.175
**Pre-pregnancy BMI (kg/m^2^)**	23.92 ± 4.31
**Pregnancy BMI increase (kg/m^2^)**	5.10 ± 1.89
**Marital status** Married/living together: Widow: Divorced/separated: Single:	314 (96.9) 1 (0.3) 1 (0.3) 8 (2.5)
**Education** None: Elementary school: Vocational school: High school: University degree:	1 (0.3) 4 (1.2) 31 (9.6) 108 (33.3) 180 (55.6)
**Socio-economic status** Employed: Unemployed: Student:	285 (88.0) 23 (7.1) 16 (4.9)
**Smoking status** Never smoked: Smoker: Ex-smoker:	221 (68.2) 45 (13.9) 58 (17.9)

**Table 3:** Cu and Zn in cord blood expressed as two standard deviations from the mean levels.

	Cu (µg/L)	Zn (µg/L)
**Week of Pregnancy**	+/−2 SD	+/−2 SD
**26–30**	32–440	914–1519
**31–32**	0–605	752–1980
**33–34**	0–584	778–1718
**35–36**	121–529	917–1647
**37–42**	159–695	823–1635

**Table 4:** Characteristics of infants and pregnancy outcomes (n = 324).

Variable	Mean ± SD or Frequency (N(%))
**Sex** Male: Female:	159 (49.1%) 165 (50.9%)
**Gestational age (weeks)**	39.52 ± 1.66
**Gestational age (categorical)** Pre-term (<37 weeks): Term (37–42 weeks): Post-term (>42 weeks):	11 (3.4%) 214 (66.0%) 99 (30.6%)
**Birth weight (g)**	3439.10 ± 486.47
**Birth length (cm)**	51.75 ± 2.30
**Size for gestational age** SGA: AGA: LGA:	23 (7.1%) 261 (80.6%) 40 (12.3%)
**Delivery type** Spontaneous vaginal: Induced with medications: Emergency Caesarean section: Vacuum: Breach/rotation:	150 (46.3%) 150 (46.3%) 19 (5.9%) 4 (1.2%) 1 (0.3%)

SGA: small for gestational age; AGA: appropriate for gestational age; LGA: large for gestational age.

## Supplementary Materials

The following supporting information can be downloaded at: https://www.mdpi.com/article/10.3390/nu14214667/s1, QuestionnaireS1: Child neurodevelopment among residents in the Mediterranean coastal regions of Italy, Slovenia, Croatia and Greece: the role of environmental exposure to heavy metals.
