# Supplementary material for: Association of Zn and Cu Levels in Cord Blood and Maternal Milk with Pregnancy Outcomes among the Slovenian Population
Source: Nutrients. 2022 Nov 4;14(21):4667. doi: 10.3390/nu14214667 (PMC9654275; doi:10.3390/nu14214667)
Supplement: Supplementary file 1 [file nutrients-14-04667-s001.zip › nutrients-1874247-supplementary.pdf]

Child neurodevelopment among residents in the Mediterranean coastal regions of Italy, Slovenia, Croatia and Greece: the role of environmental exposure to heavy metals

|__|__|__|__|v1

Date of Interview (DDMMYY) |__|__|__|__|__|__| |__|__|__|__|__|__|v2
Start Time (HHMM) |__|__|__|__| |__|__|__|__|v3

**D1**	**Full name of mother**………………………………………………………………….		|__|__|__|__|v4
**D2**	**Date of birth** (DDMMYY)	|__|__|__|__|__|__|	|__|__|__|__|__|__|v5
**D3**	**Full name of newborn**……………………………………………………………….		|__|__|__|__|v6
**D4**	**Sex of newborn**	1. Female<br>2. Male	|__|v7
**D5**	**Date of birth of newborn** (DDMMYY)	|__|__|__|__|__|__|	|__|__|__|__|__|__|v8
**D6**	**Address**<br>Street…………………………………………N°……………….<br>Municipality/Subdivision……………………Province…………………………..		|__|__|__|__|__|v9

D7 **Do you agree to participate in the study?**
1. Yes
2. No

|__|v10

PERSONAL DATA OF MOTHER AND HUSBAND/ LIVE IN MATE

D8 **Place of birth of mother**

Municipality……………………………………………………………………………… |__|__|__|__|__|v11

Province/Foreign Country………………………………………………………….

D9 **School title of mother** |__|v12

1. None
2. Elementary school
3. Middle school
4. High school
5. College degree

D10 **Name of husband/live in mate**……………………………………………….

D11 **Date of birth of husband/live in mate**(DDMMYY)**:** |__|__|__|__|__|__| |__|__|__|__|__|__|v13

D12 **School title of husband/live in mate**: |__|v14

1. None
2. Elementary school
3. Middle school
4. High school
5. College degree

D13 **Profession of husband/live in mate**:

……………………………………………………………………………………………

…………………………………………………………………………………………..

HEALTH STATUS AND OBSTETRIC/GYNAECOLOGIC HISTORY

|__|v15

D14 Was the last pregnancy your first one?

1. Yes
2. No

|__|__|v16

D15 How many previous pregnancies have you had? |__|__|

D16 What was the year and outcome of previous pregnancies?

Pregnancy	Year	Outcome
1		
2		
3		
4		
1=live born full term 2=live born premature 3=live born underweight 4=live born with malformations/genetic syndromes 5=miscarriage 6=stillbirth		

|__|__|v17 |__|v18
|__|__|v19 |__|v20
|__|__|v21 |__|v22
|__|__|v23 |__|v24

D17	Regarding the father of the child:	
A	Is he alive or deceased?	1. Alive (go to B)<br>2. Deceased (go to C, D)
B	If alive, is he in good health?	1. Yes<br>2. No: he is affected by..
C	If deceased, give age of death in years:	|__|__|
D	What was the cause of death?.......................................	

|__|v25
|__|v26
|__|__|__|__|v27
|__|__|v28
|__|__|__|__|v29

<table>
<tr><td>D18</td><td colspan="2">Regarding other children (newborn excluded):</td><td></td></tr>
<tr><td colspan="3">Child 1</td><td></td></tr>
<tr><td>A</td><td>Is he/she alive or deceased?</td><td>1. Alive (go to B)
2. Deceased (go to C, D)</td><td>|__|v30</td></tr>
<tr><td>B</td><td>If alive, is he/she in good health?</td><td>1. Yes
2. No: he/she is affected by...</td><td>|__|v31
|__|__|__|__|v32</td></tr>
<tr><td>C</td><td>If deceased, give age of death in years, months or days:</td><td>YY|__|__| MM|__|__| DD|__|__|</td><td>|__|__|__|__|__|__|v33</td></tr>
<tr><td>D</td><td colspan="2">What was the cause of death?......................................</td><td>|__|__|__|__|v34</td></tr>
</table>

<table>
<tr><td colspan="3">Child 2</td><td></td></tr>
<tr><td>A</td><td>Is he/she alive or deceased?</td><td>1. Alive (go to B)
2. Deceased (go to C, D)</td><td>|__|v35</td></tr>
<tr><td>B</td><td>If alive, is he/she in good health?</td><td>1. Yes
2. No: he/she is affected by...</td><td>|__|v36
|__|__|__|__|v37</td></tr>
<tr><td>C</td><td>If deceased, give age of death in years, months or days:</td><td>YY|__|__| MM|__|__| DD|__|__|</td><td>|__|__|__|__|__|__|v38</td></tr>
<tr><td>D</td><td colspan="2">What was the cause of death?......................................</td><td>|__|__|__|__|v39</td></tr>
</table>

<table>
<tr><td colspan="3">Child 3</td><td></td></tr>
<tr><td>A</td><td>Is he/she alive or deceased?</td><td>1. Alive (go to B)
2. Deceased (go to C, D)</td><td>|__|v40</td></tr>
<tr><td>B</td><td>If alive, is he/she in good health?</td><td>1. Yes
2. No: he/she is affected by...</td><td>|__|v41
|__|__|__|__|42</td></tr>
<tr><td>C</td><td>If deceased, give age of death in years, months or days:</td><td>YY|__|__| MM|__|__| DD|__|__|</td><td>|__|__|__|__|__|__|v43</td></tr>
<tr><td>D</td><td colspan="2">What was the cause of death?......................................</td><td>|__|__|__|__|v44</td></tr>
</table>

Child 4			
**A**	**Is he/she alive or deceased?**	1. Alive (go to B)<br>2. Deceased (go to C, D)	|__|v45
**B**	**If alive, is he/she in good health?**	1. Yes<br>2. No: he/she is affected by...	|__|v46<br>|__|__|__|__|v47
**C**	**If deceased, give age of death in years, months or days:**	YY|__|__| MM|__|__| DD|__|__|	|__|__|__|__|__|__|v48
**D**	**What was the cause of death?......................................**		|__|__|__|__|v49

D19 Is/was (if deceased) anyone in your family affected by any of the following diseases?:

	1=Yes<br>2=No	1=child's father; 2=child; 3=parents of father or mother; 4=sibling of father or mother	
Cardiovascular diseases			|__|v50<br>|__|__|__|__|v51
Neurological diseases			|__|v52<br>|__|__|__|__|v53
Genetic diseases			|__|v54<br>|__|__|__|__|v55
Tumors			|__|v56<br>|__|__|__|__|v57
Diabetes Mellitus			|__|v58<br>|__|__|__|__|v59
Hypertension			|__|v60<br>|__|__|__|__|v61

D20 Before and during pregnancy, were you affected by:

	**Before (B) or During (D) Pregnancy 2=No**		**Specify actual disease**
Diabetes			
Asthma			
Allergies			
Epilepsy			
Hypertension			
Vomiting			
Hypothyroidism			
Hyperthyroidism			
Lupus e. s.			
Rheumatoid diseases			
Urinary infections			
Infections			
Fever			
Seizure			
Anaemia			
Cardiac diseases			

|__|v62 |_|_|_|_|v63

|__|v64 |_|_|_|_|v65

|__|v66 |_|_|_|_|v67

|__|v68 |_|_|_|_|v69

|__|v70 |_|_|_|_|v71

|__|v72 |_|_|_|_|v73

|__|v74 |_|_|_|_|v75

|__|v76 |_|_|_|_|v77

|__|v78 |_|_|_|_|v79

|__|v80 |_|_|_|_|v81

|__|v82 |_|_|_|_|v83

|__|v84 |_|_|_|_|v85

|__|v86 |_|_|_|_|v87

|__|v88 |_|_|_|_|v89

|__|v90 |_|_|_|_|v91

|__|v92 |_|_|_|_|v93

D21 **Did you take any therapeutic drugs during pregnancy?**

1. Yes
2. No (go to D23)

|__|v94

D22 **If "Yes", which one(s)**

Brand Name	Reason	From	To	Times a day

|__|__|__|v95 |__|__|__|__|v96
|__|__|v97 |__|__|v98 |__|v99

|__|__|__|v100 |__|__|__|__|v101
|__|__|v102 |__|__|v103 |__|v104

|__|__|__|v105 |__|__|__|__|v106
|__|__|v107 |__|__|v108 |__|v109

|__|__|__|v110 |__|__|__|__|v111
|__|__|v112 |__|__|v113 |__|v114

|__|__|__|v115 |__|__|__|__|v116
|__|__|v117 |__|__|v118 |__|v119

|__|__|__|v120 |__|__|__|__|v121
|__|__|v122 |__|__|v123 |__|v124

D23 **How many OB/GYN exams did you undergo during pregnancy?** |__|__|

|__|__|v125

D24 **How many ultrasound exams?** |__|__|

|__|__|v126

D25 **Did you have any dental exams during pregnancy?**

1. Yes
2. No (go to D28)

|__|v127

D26	**During dental exams, did you have any tooth fillings?**	1. Yes<br>2. No	|__|v128
D27	**How many tooth fillings?**	1. < 2<br>2. 3-5<br>3. 6-9<br>4. 10+	|__|v129
D28	**What was your average weight before pregnancy?**	Kg: |__|__|	|__|__|v130
D29	**How many Kg did you gain during pregnancy?**	Kg: |__|__|	|__|__|v131
D30	**How tall are you?**	Cm: |__|__|__|	|__|__|__|v132
D31	**How tall is the father of the baby?**	Cm: |__|__|__|	|__|__|__|v133
D32	**What is the father's weight?**	Kg: |__|__|__|	|__|__|__|v134

HEALTH STATUS OF NEWBORN

D33	**How much did your baby weigh at birth?**	Gm: |__|__|__|__|	|__|__|__|__|v135
D34	**How long was your baby at birth?**	Cm: |__|__|	|__|__|v136
D35	**What type of delivery did you have?**	1. Spontaneous<br>2. Induced with drugs<br>3. Caesarean section<br>4. with suction cup<br>5. breach<br>6. with forceps	|__|v137

D36 **After birth, was your baby affected by any of the following?**

Jaundice	1. Yes<br>2. No	|__|v138
Infections	1. Yes<br>2. No	|__|v139
Respiratory problems	1. Yes<br>2. No	|__|v140
Seizures	1. Yes<br>2. No	|__|v141

D37 **Was your baby ever hospitalized?**

1. Yes
2. No (go to D38)

|__|v142

Weeks of age	Reason	Medications for treatment	
			|__|__|v143|__|__|__|__|__|v144
			|__|__|v145|__|__|__|__|__|v146
			|__|__|v147|__|__|__|__|__|v148
			|__|__|v149|__|__|__|__|__|v150
			|__|__|v151|__|__|__|__|__|v152

D38 **Hospitalizations excluded, did your baby ever need medical attention?**

1. Yes
2. No (go to D39)

|__|__|v153

D39 **Presently, how is your baby being nursed:**

1. Breast milk only
2. Mostly breast milk
3. Partially breast milk
4. Artificial milk (formula)

|__|v154

D40 **In the preceeding weeks, your baby was nursed with:**

From (weeks)	To (weeks)	Method
**Method: 1.** Breast milk only **2.** Mostly breast milk **3.** Partially breast milk **4.** Artificial milk (formula**)**		

|__|__|v155 |__|__|v156|__|v157

|__|__|v158 |__|__|v159|__|v160

|__|__|v161 |__|__|v162|__|v163

|__|__|v164 |__|__|v165|__|v166

|__|__|v1167 |__|__|v168|__|v169

|__|__|v170 |__|__|v171|__|v172

D41 **Is your baby taking the pacifier?**

1. Yes
2. No

|__|v173

D42 **Presently, where does your baby sleep?**

1. in the same room with parents
2. alone in his/her room
3. with “others”

|__|v174

MOTHER’S DIET

D43 **<u>During pregnancy</u>, with what frequency, on the average, did you eat the following foods?**

<table>
<tr><th>Food item</th><th>Daily</th><th>Weekly</th><th>Monthly</th><th>9 mos</th><th></th></tr>
<tr><td>Milk (1 glass)
Local/Home farm-raised
Store bought</td><td>|__|v175
|__|v179</td><td>|__|__|v176
|__|__|V180</td><td>|__|__|__|v177
|__|__|__|v181</td><td>|__|__|__|v178
|__|__|__|v182</td><td>175-182</td></tr>
<tr><td>Yogurt (1 jar, 125 g)</td><td>|__|v183</td><td>|__|__|v184</td><td>|__|__|__|v185</td><td>|__|__|__|v186</td><td>183-186</td></tr>
<tr><td>Pasta or rice with oil or butter (80 g)</td><td>|__|v187</td><td>|__|__|v188</td><td>|__|__|__|v189</td><td>|__|__|__|v190</td><td>187-190</td></tr>
<tr><td>Pasta or rice with tomato sauce (80 g)</td><td>|__|v191</td><td>|__|__|v192</td><td>|__|__|__|v193</td><td>|__|__|__|v194</td><td>191-194</td></tr>
<tr><td>Pasta or rice with ragù sauce/ Lasagna/cannelloni (80 g)</td><td>|__|v195</td><td>|__|__|v196</td><td>|__|__|__|v197</td><td>|__|__|__|v198</td><td>195-198</td></tr>
<tr><td>Pasta or rice with fish sauce (80 g)</td><td>|__|v199</td><td>|__|__|v200</td><td>|__|__|__|v201</td><td>|__|__|__|v202</td><td>199-202</td></tr>
<tr><td>Vegetable/legume soup/pasta with beans (250 g)</td><td>|__|v203</td><td>|__|__|v204</td><td>|__|__|__|v205</td><td>|__|__|__|v206</td><td>203-206</td></tr>
<tr><td>Fish soup/ broth (100 g)</td><td>|__|v207</td><td>|__|__|v208</td><td>|__|__|__|v209</td><td>|__|__|__|v210</td><td>207-210</td></tr>
<tr><td>Pizza (1, 200 g)</td><td>|__|v211</td><td>|__|__|v212</td><td>|__|__|__|v213</td><td>|__|__|__|v214</td><td>211-214</td></tr>
<tr><td>Bread (1 bun/slice)</td><td>|__|v215</td><td>|__|__|v216</td><td>|__|__|__|v217</td><td>|__|__|__|v218</td><td>215-218</td></tr>
<tr><td>Crackers, grissini, melba toast (1 serving/5/3)</td><td>|__|v219</td><td>|__|__|v220</td><td>|__|__|__|v221</td><td>|__|__|__|v222</td><td>219-222</td></tr>
<tr><td>Polenta (2 slices)</td><td>|__|v223</td><td>|__|__|v224</td><td>|__|__|__|v225</td><td>|__|__|__|v226</td><td>223-226</td></tr>
<tr><td>Boiled chicken/turkey, grilled (1 serv., 200 g)
Local/Home farm-raised
Store bought</td><td>|__|v227
|__|v231</td><td>|__|__|v228
|__|__|v232</td><td>|__|__|__|v229
|__|__|__|v233</td><td>|__|__|__|v230
|__|__|__|v234</td><td>227-234</td></tr>
<tr><td>Chicken/turkey roasted, stewed, fried (1 serv, 200 g)
Local/Home farm-raised
Store bought</td><td>|__|v235
|__|v239</td><td>|__|__|v236
|__|__|v240</td><td>|__|__|__|v237
|__|__|__|v241</td><td>|__|__|__|v238
|__|__|__|v242</td><td>235-242</td></tr>
<tr><td>Beef/pork boiled, grilled (1 serv., 120 g)
Local/Home farm-raised
Store bought</td><td>|__|v243
|__|v247</td><td>|__|__|v244
|__|__|v248</td><td>|__|__|__|v245
|__|__|__|v249</td><td>|__|__|__|v246
|__|__|__|v250</td><td>243-250</td></tr>
<tr><td>Beef/pork roasted, stewed, fried (1 serv., 150 g)
Local/Home farm-raised
Store bought</td><td>|__|v251
|__|v255</td><td>|__|__|v252
|__|__|v256</td><td>|__|__|__|v253
|__|__|__|v257</td><td>|__|__|__|v254
|__|__|__|v258</td><td>251-258</td></tr>
</table>

<table>
<tr><th>Food item</th><th>Daily</th><th>Weekly</th><th>Monthly</th><th>9 mos</th><th></th></tr>
<tr><td>Wild game meats
Local/Home farm raised
Store bought</td><td>
|__|v259
|__|v263</td><td>
|__|__|v260
|__|__|v264</td><td>
|__|__|__|v261
|__|__|__|v265</td><td>
|__|__|__|v262
|__|__|__|v266</td><td>259-266</td></tr>
<tr><td>Assorted fried fish (1 serv.150 g)</td><td>|__|v267</td><td>|__|__|v268</td><td>|__|__|__|v269</td><td>|__|__|__|v270</td><td>267-270</td></tr>
<tr><td>Assorted fish grilled (1 serv.150 g)</td><td>|__|v271</td><td>|__|__|v272</td><td>|__|__|__|v273</td><td>|__|__|__|v274</td><td>271-274</td></tr>
<tr><td>Prosciutto/deli meats (50 g)</td><td>|__|v275</td><td>|__|__|v276</td><td>|__|__|__|v277</td><td>|__|__|__|v278</td><td>275-278</td></tr>
<tr><td>Eggs (1)
Local/Home farm-raised
Store bought</td><td>
|__|v279
|__|v283</td><td>
|__|__|v280
|__|__|v284</td><td>
|__|__|__|v281
|__|__|__|v285</td><td>
|__|__|__|v282
|__|__|__|v286</td><td>279-286</td></tr>
<tr><td>Cheese (1 serving, 100 g)</td><td>|__|v287</td><td>|__|__|v288</td><td>|__|__|__|v289</td><td>|__|__|__|v290</td><td>287-290</td></tr>
<tr><td>Dessert/fruit tarts (1 slice or cup)</td><td>|__|v291</td><td>|__|__|v292</td><td>|__|__|__|v293</td><td>|__|__|__|v294</td><td>291-294</td></tr>
</table>

D44 **<u>During pregnancy</u>, with what frequency, on the average, did you eat the following sea foods?**

<table>
<tr><th>Food item</th><th>Daily</th><th>Weekly</th><th>Monthly</th><th>9 mos</th><th></th></tr>
<tr><td>Fish (150 g)</td><td>|__|v295</td><td>|__|__|v296</td><td>|__|__|__|v297</td><td>|__|__|__|v298</td><td>295-298</td></tr>
<tr><td>Molluscs/shellfish (150 g)</td><td>|__|v299</td><td>|__|__|v300</td><td>|__|__|__|v301</td><td>|__|__|__|v302</td><td>299-302</td></tr>
<tr><td>Tuna, mackerel, sardines packed in oil (1 can or 80 g)</td><td>|__|v303</td><td>|__|__|v304</td><td>|__|__|__|v305</td><td>|__|__|__|v306</td><td>303-306</td></tr>
</table>

D45 **<u>During pregnancy</u>, with what frequency, on the average, did you eat the following types of fish?**

<table>
<tr><th>Food item</th><th>Daily</th><th>Weekly</th><th>Monthly</th><th>9 mos</th><th>1=fresh
2=frozen
3=canned
4=don't know</th><th></th></tr>
<tr><td>Eel</td><td>|__|v307</td><td>|__|__|v308</td><td>|__|__|__|v309</td><td>|__|__|__|v310</td><td>|__|v311</td><td>307-311</td></tr>
<tr><td>Bass</td><td>|__|v312</td><td>|__|__|v313</td><td>|__|__|__|v314</td><td>|__|__|__|v315</td><td>|__|v316</td><td>312-316</td></tr>
<tr><td>Gilthead</td><td>|__|v317</td><td>|__|__|v318</td><td>|__|__|__|v319</td><td>|__|__|__|v320</td><td>|__|v321</td><td>317-321</td></tr>
<tr><td>Grey mullet</td><td>|__|v322</td><td>|__|__|v323</td><td>|__|__|__|v324</td><td>|__|__|__|v325</td><td>|__|v326</td><td>322-326</td></tr>
<tr><td>Riboni</td><td>|__|v327</td><td>|__|__|v328</td><td>|__|__|__|v329</td><td>|__|__|__|v330</td><td>|__|v331</td><td>327-331</td></tr>
<tr><td>Sole</td><td>|__|v332</td><td>|__|__|v333</td><td>|__|__|__|v334</td><td>|__|__|__|v335</td><td>|__|v336</td><td>332-336</td></tr>
<tr><td>Fresh tuna</td><td>|__|v337</td><td>|__|__|v338</td><td>|__|__|__|v339</td><td>|__|__|__|v340</td><td>|__|v341</td><td>337-341</td></tr>
<tr><td>Moli/melù</td><td>|__|v342</td><td>|__|__|v343</td><td>|__|__|__|v344</td><td>|__|__|__|v345</td><td>|__|v346</td><td>342-346</td></tr>
<tr><td>Asià</td><td>|__|v347</td><td>|__|__|v348</td><td>|__|__|__|v349</td><td>|__|__|__|v350</td><td>|__|v351</td><td>347-351</td></tr>
</table>

<table>
<tr><th>Food item</th><th>Daily</th><th>Weekly</th><th>Monthly</th><th>9 mos</th><th>1=fresh
2=frozen
3=canned
4=don’t know</th><th></th></tr>
<tr><td>Mackerels</td><td>|__|v352</td><td>|__|__|v353</td><td>|__|__|__|v354</td><td>|__|__|__|v355</td><td>|__|v356</td><td>352-356</td></tr>
<tr><td>Smooth dogfish</td><td>|__|v357</td><td>|__|__|v358</td><td>|__|__|__|v359</td><td>|__|__|__|v360</td><td>|__|v361</td><td>357-361</td></tr>
<tr><td>Plaice</td><td>|__|v362</td><td>|__|__|v363</td><td>|__|__|__|v364</td><td>|__|__|__|v365</td><td>|__|v366</td><td>362-366</td></tr>
<tr><td>Angler</td><td>|__|v367</td><td>|__|__|v368</td><td>|__|__|__|v369</td><td>|__|__|__|v370</td><td>|__|v371</td><td>367-371</td></tr>
<tr><td>Vitello-mare</td><td>|__|v372</td><td>|__|__|v373</td><td>|__|__|__|v374</td><td>|__|__|__|v375</td><td>|__|v376</td><td>372-376</td></tr>
<tr><td>p.S.Pietro</td><td>|__|v377</td><td>|__|__|v378</td><td>|__|__|__|v379</td><td>|__|__|__|v380</td><td>|__|v381</td><td>377-381</td></tr>
<tr><td>Pilchards/ Sardines</td><td>|__|v382</td><td>|__|__|v383</td><td>|__|__|__|v384</td><td>|__|__|__|v385</td><td>|__|v386</td><td>382-386</td></tr>
<tr><td>Shads</td><td>|__|v387</td><td>|__|__|v388</td><td>|__|__|__|v389</td><td>|__|__|__|v390</td><td>|__|v391</td><td>387-391</td></tr>
<tr><td>Trout</td><td>|__|v392</td><td>|__|__|v393</td><td>|__|__|__|v394</td><td>|__|__|__|v395</td><td>|__|v396</td><td>392-396</td></tr>
<tr><td>Cod</td><td>|__|v397</td><td>|__|__|v398</td><td>|__|__|__|v399</td><td>|__|__|__|v400</td><td>|__|v401</td><td>397-401</td></tr>
<tr><td>Swordfish</td><td>|__|v402</td><td>|__|__|v403</td><td>|__|__|__|v404</td><td>|__|__|__|v405</td><td>|__|v406</td><td>402-406</td></tr>
<tr><td>Salmon</td><td>|__|v407</td><td>|__|__|v408</td><td>|__|__|__|v409</td><td>|__|__|__|v410</td><td>|__|v411</td><td>407-411</td></tr>
<tr><td>Other saltwater fish (list):</td><td>|__|v412</td><td>|__|__|v413</td><td>|__|__|__|v414</td><td>|__|__|__|v415</td><td>|__|v416</td><td>412-416</td></tr>
<tr><td>Other freshwater fish (list):</td><td>|__|v417</td><td>|__|__|v418</td><td>|__|__|__|v419</td><td>|__|__|__|v420</td><td>|__|v421</td><td>417-421</td></tr>
</table>

D46 **During pregnancy, how many times, on the average did you eat fish at a restaurant?** |__|v422

1. <1/month
2. 1-3/month
3. 1/week
4. 2-4/week
5. >4/week
6. never

D47 **If breast-feeding, with what frequency do you presently eat fish?**

Food item	Daily	Weekly	Monthly	
**Fish** (150 g)	|__|v423	|__|__|v424	|__|__|__|v425	423-425
**Molluscs/shellfish** (150 g)	|__|v426	|__|__|v427	|__|__|__|v428	426-428
**Tuna, mackerel, sardines packed in oil** (1 can or 80 g)	|__|v429	|__|__|v430	|__|__|__|v431	429-431

D48 **In the winter, where do you habitually buy <u>fresh fish</u>** (2 mostly used): |__|v432

1. Fish-shop
2. Fish market
3. Grocery store
4. From the fisherman
5. Fishes on her own
6. Other:..................
9. Does not buy fresh fish

D49 **Can you indicate the name and/or the Municipality where you habitually buy fish in the <u>winter</u>?**

Name .... |__|__|__|v433

Municipality .... |__|__|__|__|__|v434

Name .... |__|__|__|v435

Municipality .... |__|__|__|__|__|v436

D50 **In the summer, where do you habitually buy <u>fresh fish</u>** (2 mostly used): |__|v437

1. Fish-shop
2. Fish market
3. Grocery store
4. From the fisherman
5. Fishes on her own
6. Other:............................
9. Does not buy fresh fish

D51 **Can you indicate the name and/or the Municipality where you habitually buy fish in the summer?**

**Name**	....	|__|__|__|v438
**Municipality**	....	|__|__|__|__|__|v439
**Name**	....	|__|__|__|v440
**Municipality**	....	|__|__|__|__|__|v441

D52 **Do you buy pre-packaged fish?**

1. Yes
2. No

|__|v442

D53 **<u>During pregnancy</u>, how many times, on the average, did you consume the following vegetables?**

Food Item	Raw			Cooked			In season (n° of months)
	Daily	Weekly	Monthly	Daily	Weekly	Monthly	
**Potatoes** (1;150g)<br>Local/Home garden<br>Store bought	|__|v443<br>|__|v444	|__|__|v445<br>|__|__|v446	|__|__|__|v447<br>|__|__|__|v448	|__|v449<br>|__|v450	|__|__|v451<br>|__|__|v452	|__|__|__|v453<br>|__|__|__|v454	|__|__|v455<br>|__|__|v456
**Mixed salad** (tomatoes, cucumbers, carrots) (50 g)<br>Local/Home garden<br>Store bought	|__|v457<br>|__|v458	|__|__|v459<br>|__|__|v460	|__|__|__|v461<br>|__|__|__|v462	|__|v463<br>|__|v464	|__|__|v465<br>|__|__|v466	|__|__|__|v467<br>|__|__|__|v468	|__|__|v469<br>|__|__|v470
**Green and red lattuce** (50g)<br>Local/Home garden<br>Store bought	|__|v471<br>|__|v472	|__|__|v473<br>|__|__|v474	|__|__|__|v475<br>|__|__|__|v476	|__|v477<br>|__|v478	|__|__|v479<br>|__|__|v480	|__|__|__|v481<br>|__|__|__|v482	|__|__|v483<br>|__|__|v484
**Spinach/ Chard** (200g)<br>Local/Home garden<br>Store bought	|__|v485<br>|__|v486	|__|__|v487<br>|__|__|v488	|__|__|__|v489<br>|__|__|__|v490	|__|v491<br>|__|v492	|__|__|v493<br>|__|__|v494	|__|__|__|v495<br>|__|__|__|v496	|__|__|v497<br>|__|__|v498
**Cabbage, Cauliflower, Broccoli, Brussels sprouts, Savoy cabbage, turnips** (125g)<br>Local/Home garden<br>Store bought	|__|v499<br>|__|v500	|__|__|v501<br>|__|__|v502	|__|__|__|v503<br>|__|__|__|v504	|__|v505<br>|__|v506	|__|__|v507<br>|__|__|v508	|__|__|__|v509<br>|__|__|__|v510	|__|__|v511<br>|__|__|v512

<table>
<tr><th rowspan="2">FOOD ITEM</th><th colspan="3">Raw</th><th colspan="3">Cooked</th><th>In season(n° of months)</th></tr>
<tr><th>Daily</th><th>Weekly</th><th>Monthly</th><th>Daily</th><th>Weekly</th><th>Monthly</th><th></th></tr>
<tr><td>Carrots (100g)
<u>Local/Home garden</u>
<u>Store bought</u></td><td>|__|v513
|__|v514</td><td>|__|__|v515
|__|__|v516</td><td>|__|__|__|v517
|__|__|__|v518</td><td>|__|v519
|__|v520</td><td>|__|__|v521
|__|__|v522</td><td>|__|__|__|v523
|__|__|__|v524</td><td>|__|__|v525
|__|__|v526</td></tr>
<tr><td>Fresh tomatoes (150g)
<u>Local/Home garden</u>
<u>Store bought</u></td><td>|__|v527
|__|v528</td><td>|__|__|v529
|__|__|v530</td><td>|__|__|__|v531
|__|__|__|v532</td><td>|__|v533
|__|v534</td><td>|__|__|v535
|__|__|v536</td><td>|__|__|__|v537
|__|__|__|v538</td><td>|__|__|v539
|__|__|v540</td></tr>
<tr><td>Peppers, Zucchini, Egg-plant, winter squash (150g)
<u>Local/Home garden</u>
<u>Store bought</u></td><td>|__|v541
|__|v542</td><td>|__|__|v543
|__|__|v544</td><td>|__|__|__|v544
|__|__|__|v545</td><td>|__|v546
|__|v547</td><td>|__|__|v548
|__|__|v549</td><td>|__|__|__|v550
|__|__|__|v551</td><td>|__|__|v552
|__|__|v553</td></tr>
<tr><td>Artichokes (1 whole, 3 bottoms)
<u>Local/Home garden</u>
<u>Store bought</u></td><td>|__|v554
|__|v555</td><td>|__|__|v556
|__|__|v557</td><td>|__|__|__|v558
|__|__|__|v559</td><td>|__|v560
|__|v561</td><td>|__|__|v562
|__|__|v563</td><td>|__|__|__|v564
|__|__|__|v565</td><td>|__|__|v566
|__|__|v567</td></tr>
<tr><td>Fennel (1;100g)
<u>Local/Home garden</u>
<u>Store bought</u></td><td>|__|v568
|__|v569</td><td>|__|__|v570
|__|__|v571</td><td>|__|__|__|v572
|__|__|__|v573</td><td>|__|v574
|__|v575</td><td>|__|__|v576
|__|__|v577</td><td>|__|__|__|v578
|__|__|__|v579</td><td>|__|__|v580
|__|__|v581</td></tr>
<tr><td>Peas, beans, green beans, chickpeas, lentils (100g)
<u>Local/Home garden</u>
<u>Store bought</u></td><td>|__|v582
|__|v583</td><td>|__|__|v584
|__|__|v585</td><td>|__|__|__|v586
|__|__|__|v587</td><td>|__|v588
|__|v589</td><td>|__|__|v590
|__|__|v591</td><td>|__|__|__|v592
|__|__|__|v593</td><td>|__|__|v594
|__|__|v595</td></tr>
</table>

D54	**During pregnancy, how many times, on the average, did you consume the following fruits?**

<table>
<tr><th>FOOD ITEM</th><th>Daily</th><th>Weekly</th><th>Monthly</th><th>In Season
(n° of months)</th><th></th></tr>
<tr><td>Bananas (1)</td><td>|__|v596</td><td>|__|__|v597</td><td>|__|__|__|v598</td><td>|__||__|v599</td><td>596-599</td></tr>
<tr><td>Apples or pears (1)
Local/Home garden
Store bought</td><td>|__|v600
|__|v601</td><td>|__|__|v602
|__|__|v603</td><td>|__|__|__|v604
|__|__|__|v605</td><td>|__|__|v606
|__|__|v607</td><td>600-607</td></tr>
<tr><td>Peaches (1) /apricots (3) /prunes (3), (100 g)
Local/Home garden
Store bought</td><td>|__|v608
|__|v609</td><td>|__|__|v610
|__|__|v611</td><td>|__|__|__|v612
|__|__|__|v613</td><td>|__|__|v614
|__|__|v615</td><td>608-615</td></tr>
<tr><td>Cantaloupe(2 slices)/watermelon (1 slice)
Local/Home garden
Store bought</td><td>|__|v616
|__|v617</td><td>|__|__|v618
|__|__|v619</td><td>|__|__|__|v620
|__|__|__|v621</td><td>|__|__|v622
|__|__|v623</td><td>616-623</td></tr>
<tr><td>Strawberries/cherries (1 cup, 150 g)
Local/Home garden
Store bought</td><td>|__|v624
|__|v625</td><td>|__|__|v626
|__|__|v627</td><td>|__|__|__|v628
|__|__|__|v629</td><td>|__|__|v630
|__|__|v631</td><td>624-631</td></tr>
<tr><td>Oranges (1)/ grapefruits(1)/ lemons (1) tangerines (2)/ fruit squeezes (150 g)
Local/Home garden
Store bought</td><td>|__|v632
|__|v633</td><td>|__|__|v634
|__|__|v635</td><td>|__|__|__|v636
|__|__|__|v637</td><td>|__|__|v638
|__|__|v639</td><td>632-639</td></tr>
<tr><td>Grape
Local/Home garden
Store bought</td><td>|__|v640
|__|v641</td><td>|__|__|v642
|__|__|v643</td><td>|__|__|__|v644
|__|__|__|v645</td><td>|__|__|v646
|__|__|v647</td><td>640-647</td></tr>
<tr><td>Kiwi
Local/Home garden
Store bought</td><td>|__|v648
|__|v649</td><td>|__|__|v650
|__|__|v651</td><td>|__|__|__|v652
|__|__|__|v653</td><td>|__|__|v654
|__|__|v655</td><td>648-655</td></tr>
<tr><td>Fruit juices (1 bottle, 125 g)</td><td>|__|v656</td><td>|__|__|v657</td><td>|__|__|__|v658</td><td>|__||__|v659</td><td>656-659</td></tr>
<tr><td>Dried fruit (50 g)</td><td>|__|v660</td><td>|__|__|v661</td><td>|__|__|__|v662</td><td>|__||__|v663</td><td>660-663</td></tr>
<tr><td>Cooked fruit (1 serving, 150 g)
Home prepared
Store bought</td><td>|__|v664
|__|v665</td><td>|__|__|v666
|__|__|v667</td><td>|__|__|__|v668
|__|__|__|v669</td><td>|__|__|v670
|__|__|v671</td><td>664-671</td></tr>
<tr><td>Berries (blueberries, raspberries, currants, 1 cup)
Local/Home garden
Store bought</td><td>|__|v672
|__|v673</td><td>|__|__|v674
|__|__|v675</td><td>|__|__|__|v676
|__|__|__|v677</td><td>|__|__|v678
|__|__|v679</td><td>672-679</td></tr>
</table>

D55 **For fruits that can be peeled or eaten whole (apples, pears, etc), do you eat them peeled or whole?**

1. Peeled
2. Whole

|__|v680

D56 **What type of seasoning/sweeteners do you use?** (use the codes reported below):

To season raw vegetables	|__|__| |__|__|	|__|__|v681 |__|__|v682
To cook or season cooked vegetables	|__|__| |__|__|	|__|__|v683 |__|__|v684
To cook meat	|__|__| |__|__|	|__|__|v685 |__|__|v686
To cook fish	|__|__| |__|__|	|__|__|v687 |__|__|v688
To season pasta or rice	|__|__| |__|__|	|__|__|v689 |__|__|v690

1=none	2=olive oil (extra pure)	3=olive oil
4=peanut oil	5=cornflower oil	6=sunflower oil
7=soy oil	8=Mixed seeds oil	9=vegetable margarine
10=butter	11=cream, besciamella, lard, shortening	12=unspecified

D57 **Did you chew gum during pregnancy?**

1. Never
2. Sometimes
3. Often
4. Do not know

|__|v691

D58 **Do you presently chew gum?**

1. Never
2. Sometimes
3. Often
4. Do not know

|__|v692

D59 During pregnancy did you take vitamins or supplements?

Substance	Brand Name	When started (MM/YY)	When stopped (MM/YY)	Times/day
Iron				
Folic acid				
Vit B				
Vit C				
Vit E				
Vit A				
Multivitamins				
Selenium				
Zinc				
Calcium				

|__|__|__|__|v693
|__|__|__|__|v694
|__|v695

|__|__|__|__|v696
|__|__|__|__|v697
|__|v698

|__|__|__|__|v699
|__|__|__|__|v700
|__|v701

|__|__|__|__|v702
|__|__|__|__|v703
|__|v704

|__|__|__|__|v705
|__|__|__|__|v706
|__|v707

|__|__|__|__|v708
|__|__|__|__|v709
|__|v710

|__|__|__|__|v711
|__|__|__|__|v712
|__|v713

|__|__|__|__|v714
|__|__|__|__|v715
|__|v716

|__|__|__|__|v717
|__|__|__|__|v718
|__|v719

|__|__|__|__|v720
|__|__|__|__|v721
|__|v722

ALCOHOLIC DRINKS AND OTHER BEVERAGES

D60 **Do you consume alcoholic drinks?**

1. Never (go to D63)
2. Yes
3. In the past

|__|v723

D61 **When do you habitually consume alcoholic drinks?**

1. During or immediately prior to meals
2. Between meals
3. Both

|__|v724

D62 **How many times a week do/did you consume the following beverages?**

	Before Pregnancy	During pregnancy	Presently	
**Wine** (125ml)				
Red	|__|__|v725	|__|__|v727	|__|__|v729	725-730
White	|__|__|v726	|__|__|v728	|__|__|v730	
**Beer** (330ml)	|__|__|v731	|__|__|v732	|__|__|v733	731-733
**Liquor** (30ml)	|__|__|v734	|__|__|v735	|__|__|v736	734-736

D63 **During pregnancy, what type of water did you habitually drink?**

1. Faucet water
2. Well or tank water
3. Bottled

|__|v737

D64 **During pregnancy, how many cups of coffee did you habitually drink?**

	Daily	Weekly	Monthly	
**Coffee**	|__|v738	|__|__|v739	|__|__|v740	738-740
**Decaf.**	|__|v741	|__|__|v742	|__|__|v743	741-743
**Tea**	|__|v744	|__|__|v745	|__|__|v746	744-746
**Decaf. tea**	|__|v747	|__|__|v748	|__|__|v749	747-749
**Herbal tea**				
Local/Home grown	|__|v750	|__|__|v752	|__|__|v754	750-755
Store bought	|__|v751	|__|__|v753	|__|__|v755	

SMOKING

D65 Presently you are a:

1. never smoker (go to D67)
2. smoker
3. ex-smoker: quit before last pregnancy
4. ex-smoker: quit during or after last pregnancy

|__|v756

D66 At what age did you start smoking? |__|__|

|__|__|v757

D67 On average, how much do/di you smoke daily?

	Before Pregnancy	During pregnancy	Presently	
Cigarettes	|__|__|v758	|__|__|v759	|__|__|v760	758-760
Cigars	|__|__|v761	|__|__|v762	|__|__|v763	761-763
Pipe	|__|__|v764	|__|__|v765	|__|__|v766	764-766

D68 Did you quit smoking during pregnancy?

1. Yes,
   If yes, when? |__|__|MM
2. No

|__|v767
|__|__|v768

D69 If an ex-smoker, at what age did you quit? |__|__|

|__|__|v769

D70 Which of the following situations do you identify with?

	Before Pregnancy	During pregnancy	Presently	
Share a home with smokers	1. Yes<br>2. No	1. Yes<br>2. No	1. Yes<br>2. No	|__|v770|__|v771<br>|__|v772
Work in an environment where everyone smokes	1. Yes<br>2. No	1. Yes<br>2. No	1. Yes<br>2. No	|__|v773|__|v774<br>|__|v775

RESIDENTIAL HISTORY

D71	Please describe your present home :

**Address**<br>Street…………………………………………N°………………<br>Municipality/Subdivision……………………Province…………………………..		|__|__|__|__|__|__|v775
**How long have you lived at the present address?**	**Since:** |__|__|__|__|MMYY	|__|__|__|__|v776
**How many homes are in the same building?**	|__|__|__|	|__|__|__|v777
**How many homes are located on the same floor?**	|__|__|	|__|__|v778
**Which floor is your home situated on?**	|__|__|	|__|__|v779
**Are you the owner or do you rent?**	1. Own<br>2. Rent	|__|v780
**How many rooms (total) are there in the home?**	|__|__|	|__|__|v781
**What is the average number of persons living in your home?**	|__|__|	|__|__|v782
**In what part of town is your home located?**	1. Center<br>2. Periphery/suburb<br>3. Rural area	|__|v783
**Your home is< 1 km from:**		
Businesses (list)	1. Yes (list)<br>……………………………………………<br>……………………………………………<br>2. No	|__|v784
Highway	1. Yes<br>2. No	|__|v785
State road	1. Yes<br>2. No	|__|v786
Provincial/County Road	1. Yes<br>2. No	|__|v787
Other high traffic roads	1. Yes<br>2. No	|__|v788
Train station	1. Yes<br>2. No	|__|v789
**From your home, have you ever noted odors from the following sources?**		
Agriculture	1. Never<br>2. Occasionally<br>3. Often	|__|v790
Industry		|__|v791

**D72**	**<u>During the last 5 years</u>, have you ever moved?**	1. Yes<br>2. No	I__I v792

**If yes, please describe your previous homes** (most recent first)		
**N°1**		
**Address**<br>Street…………………………………………N°………………<br>Municipality/Subdivision……………………Province…………………………..		I__I__I__I__I__I v793
**How long did you live at the above address?**	**From:** I__I__I__I__I MMYY<br>**To:** I__I__I__I__I MMYY	I__I__I__I__I v794<br>I__I__I__I__I v795
**How many homes were in the same building?**	I__I__I__I	I__I__I__I v796
**How many homes were located on the same floor?**	I__I__I	I__I__I v797
**Which floor was your home situated on?**	I__I__I	I__I__I v798
**Were you the owner or did you rent?**	1. Own<br>2. Rent	I__I v799
**How many rooms (total) were there in the home?**	I__I__I	I__I__I v800
**What was the average number of persons living in your home?**	I__I__I	I__I__I v801
**In what part of town was your home located?**	1. Center<br>2. Periphery/suburb<br>3. Rural area	I__I v802
**Your home was< 1 km from:**		
Businesses (list)	1. Yes (list)<br>……………………………………………<br>……………………………………………<br>2. No	I__I v803
Highway	1. Yes<br>2. No	I__I v804
State road	1. Yes<br>2. No	I__I v805
Provincial/County Road	1. Yes<br>2. No	I__I v806
Other high traffic roads	1. Yes<br>2. No	I__I v807
Train station	1. Yes<br>2. No	I__I v808
**From your home, did you ever noted odours from the following sources?**		
Agriculture	1. Never<br>2. Occasionally<br>3. Often	I__I v809
Industry		I__I v810

<table>
<tr><td colspan="2">N°2</td><td></td></tr>
<tr><td colspan="2">Address
Street………………………………………..N°……………….
Municipality/Subdivision…………………….Province………………………….</td><td>|__|__|__|__|__|__|v811</td></tr>
<tr><td>How long did you live at the above address?</td><td>From: |__|__|__|__|MMYY
To: |__|__|__|__| MMYY</td><td>|__|__|__|__|v812
|__|__|__|__|v813</td></tr>
<tr><td>How many homes were in the same building?</td><td>|__|__|__|</td><td>|__|__|__|v814</td></tr>
<tr><td>How many homes were located on the same floor?</td><td>|__|__|</td><td>|__|__|v815</td></tr>
<tr><td>Which floor was your home situated on?</td><td>|__|__|</td><td>|__|__|v816</td></tr>
<tr><td>Were you the owner or did you rent?</td><td>1. Own
2. Rent</td><td>|__|v817</td></tr>
<tr><td>How many rooms (total) were there in the home?</td><td>|__|__|</td><td>|__|__|v818</td></tr>
<tr><td>What was the average number of persons living in your home?</td><td>|__|__|</td><td>|__|__|v819</td></tr>
<tr><td>In what part of town was your home located?</td><td>1. Center
2. Periphery/suburb
3. Rural area</td><td>|__|v820</td></tr>
<tr><td colspan="2">Your home was< 1 km from:</td><td></td></tr>
<tr><td>Businesses (list)</td><td>1. Yes (list)
……………………………………………
……………………………………………
2. No</td><td>|__|v821</td></tr>
<tr><td>Highway</td><td>1. Yes
2. No</td><td>|__|v822</td></tr>
<tr><td>State road</td><td>1. Yes
2. No</td><td>|__|v823</td></tr>
<tr><td>Provincial/County Road</td><td>1. Yes
2. No</td><td>|__|v824</td></tr>
<tr><td>Other high traffic roads</td><td>1. Yes
2. No</td><td>|__|v825</td></tr>
<tr><td>Train station</td><td>1. Yes
2. No</td><td>|__|v826</td></tr>
<tr><td colspan="2">From your home, did you ever noted odours from the following sources?</td><td></td></tr>
<tr><td>Agriculture</td><td rowspan="2">1. Never
2. Occasionally
3. Often</td><td>|__|v827</td></tr>
<tr><td>Industry</td><td>|__|v828</td></tr>
</table>

**N°3**		
**Address**<br>Street……………………………………….N°……………….<br>Municipality/Subdivision…………………….Province…………………………..		
**How long did you live at the above address?**	**From:** |__|__|__|__|MMYY<br>**To:** |__|__|__|__| MMYY	|__|__|__|__|__|v829
**How many homes were in the same building?**	|__|__|__|	|__|__|__|__|v830<br>|__|__|__|__|v831<br>|__|__|__|v832
**How many homes were located on the same floor?**	|__|__|	|__|__|v833
**Which floor was your home situated on?**	|__|__|	|__|__|v834
**Were you the owner or did you rent?**	1. Own<br>2. Rent	|__|v835
**How many rooms (total) were there in the home?**	|__|__|	|__|__|v836
**What was the average number of persons living in your home?**	|__|__|	|__|__|v837
**In what part of town was your home located?**	1. Center<br>2. Periphery/suburb<br>3. Rural area	|__|v838
**Your home was< 1 km from:**		
Businesses (list)	1. Yes (list)<br>……………………………………………<br>……………………………………………<br>2. No	|__|v839
Highway	1. Yes<br>2. No	|__|v840
State road	1. Yes<br>2. No	|__|v841
Provincial/County Road	1. Yes<br>2. No	|__|v842
Other high traffic roads	1. Yes<br>2. No	|__|v843
Train station	1. Yes<br>2. No	|__|v844
**From your home, did you ever noted odours from the following sources?**		
Agriculture	1. Never<br>2. Occasionally<br>3. Often	|__|v845
Industry		|__|v846

<table>
<tr><td colspan="2">N°4</td><td></td></tr>
<tr><td colspan="2">Address
Street…………………………………………N°………………
Municipality/Subdivision…………………….Province…………………………..</td><td></td></tr>
<tr><td>How long did you live at the above address?</td><td>From: |__|__|__|__|MMYY
To: |__|__|__|__| MMYY</td><td>|__|__|__|__|__|v847
|__|__|__|__|v848</td></tr>
<tr><td>How many homes were in the same building?</td><td>|__|__|__|</td><td>|__|__|__|__|v849
|__|__|__|v850</td></tr>
<tr><td>How many homes were located on the same floor?</td><td>|__|__|</td><td>|__|__|v851</td></tr>
<tr><td>Which floor was your home situated on?</td><td>|__|__|</td><td>|__|__|v852</td></tr>
<tr><td>Were you the owner or did you rent?</td><td>1. Own
2. Rent</td><td>|__|v853</td></tr>
<tr><td>How many rooms (total) were there in the home?</td><td>|__|__|</td><td>|__|__|v854</td></tr>
<tr><td>What was the average number of persons living in your home?</td><td>|__|__|</td><td>|__|__|v855</td></tr>
<tr><td>In what part of town was your home located?</td><td>1. Center
2. Periphery/suburb
3. Rural area</td><td>|__|v856</td></tr>
<tr><td colspan="2">Your home was< 1 km from:</td><td></td></tr>
<tr><td>Businesses (list)</td><td>1. Yes (list)
……………………………………………
……………………………………………
2. No</td><td>|__|v857</td></tr>
<tr><td>Highway</td><td>1. Yes
2. No</td><td>|__|v858</td></tr>
<tr><td>State road</td><td>1. Yes
2. No</td><td>|__|v859</td></tr>
<tr><td>Provincial/County Road</td><td>1. Yes
2. No</td><td>|__|v860</td></tr>
<tr><td>Other high traffic roads</td><td>1. Yes
2. No</td><td>|__|v861</td></tr>
<tr><td>Train station</td><td>1. Yes
2. No</td><td>|__|v862</td></tr>
<tr><td colspan="2">From your home, did you ever noted odours from the following sources?</td><td></td></tr>
<tr><td>Agriculture</td><td rowspan="2">1. Never
2. Occasionally
3. Often</td><td>|__|v863</td></tr>
<tr><td>Industry</td><td>|__|v864</td></tr>
</table>

<table>
<tr><td colspan="2">N°5</td><td></td></tr>
<tr><td colspan="2">Address
Street………………………………………….N°……………….
Municipality/Subdivision…………………….Province…………………………..</td><td>|__|__|__|__|__|__|v865</td></tr>
<tr><td>How long did you live at the above address?</td><td>From: |__|__|__|__|MMYY
To: |__|__|__|__| MMYY</td><td>|__|__|__|__|v866
|__|__|__|__|v867</td></tr>
<tr><td>How many homes were in the same building?</td><td>|__|__|__|</td><td>|__|__|__|v868</td></tr>
<tr><td>How many homes were located on the same floor?</td><td>|__|__|</td><td>|__|__|v869</td></tr>
<tr><td>Which floor was your home situated on?</td><td>|__|__|</td><td>|__|__|v870</td></tr>
<tr><td>Were you the owner or did you rent?</td><td>1. Own
2. Rent</td><td>|__|v871</td></tr>
<tr><td>How many rooms (total) were there in the home?</td><td>|__|__|</td><td>|__|__|v872</td></tr>
<tr><td>What was the average number of persons living in your home?</td><td>|__|__|</td><td>|__|__|v873</td></tr>
<tr><td>In what part of town was your home located?</td><td>1. Center
2. Periphery/suburb
3. Rural area</td><td>|__|v874</td></tr>
<tr><td colspan="2">Your home was< 1 km from:</td><td></td></tr>
<tr><td>Businesses (list)</td><td>1. Yes (list)
……………………………………………
……………………………………………
2. No</td><td>|__|v875</td></tr>
<tr><td>Highway</td><td>1. Yes
2. No</td><td>|__|v876</td></tr>
<tr><td>State road</td><td>1. Yes
2. No</td><td>|__|v877</td></tr>
<tr><td>Provincial/County Road</td><td>1. Yes
2. No</td><td>|__|v878</td></tr>
<tr><td>Other high traffic roads</td><td>1. Yes
2. No</td><td>|__|v879</td></tr>
<tr><td>Train station</td><td>1. Yes
2. No</td><td>|__|v880</td></tr>
<tr><td colspan="2">From your home, did you ever noted odours from the following sources?</td><td></td></tr>
<tr><td>Agriculture</td><td rowspan="2">1. Never
2. Occasionally
3. Often</td><td>|__|v881</td></tr>
<tr><td>Industry</td><td>|__|v882</td></tr>
</table>

OCCUPATIONAL HISTORY OF MOTHER

**D73**	**Presently you are:**	1. employed, on maternity leave/leave of absence*<br>2. employed*<br>3. unemployed<br>4. housewife<br>5. student<br>6. quit working<br>7. other:(specify)…………......................<br>*If employed, go to D75*	|__|v883
**D74**	**In the last 2 years, have you been employed at least once?**	1. Yes<br>2. No (end of interview, indicate time)	|__|v884
**D75**	**At which month of pregnancy did you leave work?**	|__|__|	|__|__|v885
**D76**	**In the last 2 years, have you been employed at least once?**	1. Yes<br>2. No (end of interview, indicate time)	|__|v886
**D77**	**Have you gone back to work after pregnancy?**	1. Yes<br>2. No (go to D)	|__|v887
**D78**	**If "Yes", how long after childbirth?**	|__|__|MM<br>and/or<br>|__|__|weeks	|__|__|v888<br>|__|__|v889
**D79**	**What are (were) your major working activities?**	………………………………………………………………<br>………………………………………………………………	
**D80**	**Type of work:**	1. Employed, not manual<br>2. Employed, manual work<br>3. Self employed	|__|v890

D81 What was/is the main activity of your company, firm, office, etc.?

Activity:………………………………………………………………………………………………………. |__|__|__|__|v891

Name:………………………………………………………………………………………………………..

Municipality:……………………………………………………Prov/Country:…………………………… |__|__|__|__|__|__|v892

**D82**	**How long have you been employed by this company, firm, office, etc.?**	**From:** |__|__|__|__|MMYY<br>**To:** |__|__|__|__|MMYY	|__|__|__|__|v893<br>|__|__|__|__|v894
**D83**	**Do you (did you) hold a second job?**	1. Yes<br>Specify:...........................<br>2. No	|__|v895
**D84**	**In the past, have you held other jobs?**	1. Yes<br>2. No (end of interview, indicate time)	|__|v896

Please describe other working activities have you been involved in for longer than 6 months (most recent first):

1° PREVIOUS WORK

D85 **What were your main work activities?**

....
....
....

**D86**	**Type of work:**	1. Employed, not manual<br>2. Employed, manual work<br>3. Self employed	|__|v897

D87 **What was/is the main activity of your company, firm, office, etc.?**

Activity:.. |__|__|__|__|v898

Name:...

Municipality:...Prov/Country:................................ |__|__|__|__|__|v899

**D82**	**How long were you employed by this company, firm, office, etc.?**	**From:** |__|__|__|__|MMYY<br>**To:** |__|__|__|__|MMYY	|__|__|__|__|v900<br>|__|__|__|__|v901

2° PREVIOUS WORK

D85 What were your main work activities?

………
………
………

D86 Type of work:
1. Employed, not manual
2. Employed, manual work
3. Self employed

|__|v897

D87 What was/is the main activity of your company, firm, office, etc.?

Activity:…………………………………………………………………………………………………… |__|__|__|__|v898

Name:……………………………………………………………………………………………………

Municipality:…………………………………………Prov/Country:…………………………… |__|__|__|__|__|v899

D82 How long were you employed by this company, firm, office, etc.?

From: |__|__|__|__|MMYY |__|__|__|__|v900
To: |__|__|__|__|MMYY |__|__|__|__|v901

3° PREVIOUS WORK

D85 What were your main work activities?

………
………
………

D86 Type of work:
1. Employed, not manual
2. Employed, manual work
3. Self employed

|__|v897

D87 What was/is the main activity of your company, firm, office, etc.?

Activity:…………………………………………………………………………………………………… |__|__|__|__|v898

Name:……………………………………………………………………………………………………

Municipality:…………………………………………Prov/Country:…………………………… |__|__|__|__|__|v899

D82 How long were you employed by this company, firm, office, etc.?

From: |__|__|__|__|MMYY |__|__|__|__|v900
To: |__|__|__|__|MMYY |__|__|__|__|v901

End of Interview, time |__|__|hour |__|__|minutes |__|__|__|__|v902
